# HilE is required for synergistic activation of SPI-1 gene expression in *Salmonella enterica* serovar Typhimurium

**DOI:** 10.1186/s12866-021-02110-8

**Published:** 2021-02-16

**Authors:** Selwan Hamed, Riham M. Shawky, Mohamed Emara, James M. Slauch, Christopher V. Rao

**Affiliations:** 1 Department of Chemical and Biomolecular Engineering, University of Illinois at Urbana-Champaign, 600 S. Mathews Ave, Urbana, IL 61801 USA; 2 Department of Microbiology and Immunology, Faculty of Pharmacy, Helwan University − Ain Helwan, Helwan, 11795 Egypt; 3 Department of Microbiology, University of Illinois at Urbana-Champaign, Urbana, IL 61801 USA

**Keywords:** *Salmonell*a, SPI-1, HilE, Gene regulation, Acetate

## Abstract

**Background:**

*Salmonella enterica* serovar Typhimurium is an intestinal pathogen capable of infecting a wide range of animals. It initiates infection by invading intestinal epithelial cells using a type III secretion system encoded within *Salmonella* pathogenicity island 1 (SPI-1). The SPI-1 genes are regulated by multiple interacting transcription factors. The master regulator is HilD. HilE represses SPI-1 gene expression by binding HilD and preventing it from activating its target promoters. Previous work found that acetate and nutrients synergistically induce SPI-1 gene expression. In the present study, we investigated the role of HilE, nominally a repressor of SPI-1 gene expression, in mediating this response to acetate and nutrients.

**Results:**

HilE is necessary for activation of SPI-1 gene expression by acetate and nutrients. In mutants lacking *hilE*, acetate and nutrients no longer increase SPI-1 gene expression but rather repress it. This puzzling response is not due to the BarA/SirA two component system, which governs the response to acetate. To identify the mechanism, we profiled gene expression using RNAseq in the wild type and a Δ*hilE* mutant under different growth conditions. Analysis of these data suggested that the Rcs system, which regulates gene expression in response to envelope stress, is involved. Consistent with this hypothesis, acetate and nutrients were able to induce SPI-1 gene expression in mutants lacking *hilE* and the Rcs system.

**Conclusions:**

While the exact mechanism is unknown, these results demonstrate the HilE, nominally a repressor of SPI-1 gene expression, can also function as an activator under the growth conditions investigated. Collectively, these results provide new insights regarding SPI-1 gene regulation and demonstrate that HilE is more complex than initially envisioned.

**Supplementary Information:**

The online version contains supplementary material available at 10.1186/s12866-021-02110-8.

## Background

*Salmonella enterica* serovar Typhimurium initiates infection by invading intestinal epithelial cells using a type III secretion system encoded within *Salmonella* pathogenicity island 1 (SPI-1) [1]. Expression of the SPI-1 genes is tightly regulated by multiple transcription factors, ensuring that these genes are expressed at the right time and place using extracellular metabolites and environmental signals as sensory cues [2, 3]. HilA activates expression of the genes encoding the type III secretion system and associated effector proteins [4]. HilA expression is positively regulated by three transcription factors: HilD, HilC, and RtsA [5–8]. These three regulators can independently activate *hilA* expression. In addition, they also positively regulate their own and each other’s expression. Among the three, HilD is the most significant. In mutants lacking *hilD*, *hilA* expression is weak. In addition to these positive regulators, there is also a negative regulator, HilE [9]. This protein binds HilD and prevents it from binding its target promoters and activating transcription [10–12]. In addition to these SPI-1 specific regulators, a number of additional factors regulate SPI-1 gene expression, most by altering the expression of HilD (cf. [3]).

In previous work, we found that acetate and nutrients, provided as yeast extract, synergistically induce SPI-1 gene expression [13]. Both acetate and yeast extract are individually capable of inducing SPI-1 gene expression. However, expression is far greater when both are present in the growth medium than would be expected from their individual contributions.

As a brief background, acetate is the most abundant short-chain fatty acid in the distal small intestine [14], the site of invasion, and *Salmonella* presumably uses this metabolite to know when it is within the small intestine. Acetate is known to induce SPI-1 gene expression via the BarA/SirA two-component system [14–16]. Yeast extract, on the other hand, is complex mixture of nutrients. It is known to induce flagellar gene expression via RflP (also known as YdiV) [17–19], though induction does not appear to result from a specific metabolite but rather from improved growth resulting these nutrients [17]. Its role in invasion is unknown but nonetheless provide a chemical signal for tuning both SPI-1 and flagellar gene expression. Most likely, it provides a surrogate for other metabolite present in the distal small intestine.

Synergistic activation of SP1–1 gene expression by acetate and yeast extract results from transcriptional crosstalk with the flagellar genes [13], where expression of the flagellar regulator FliZ induces SPI-1 gene expression by post-translationally activating HilD [20] and HilD induces expression of the flagellar genes by activating the expression of the flagellar master operon *flhDC* [21].

In this work, we investigated the role of HilE in mediating synergistic induction of SPI-1 gene expression by acetate and yeast extract. We found that HilE is necessary for acetate and yeast extract to increase *hilA* expression. In mutants lacking HilE, acetate and yeast extract repressed *hilA* expression. To better understand the governing mechanism, we explored a number of regulatory mutants and also profiled gene expression using RNA-Seq. Our findings indicate that the Rcs system, involved in regulating the response to envelope stress, inhibits the activation of SPI-1 gene expression by acetate and yeast extract in mutants lacking HilE. These results further our understanding of SPI-1 gene regulation and also demonstrate that HilE has roles in addition to being a repressor of SPI-1 gene expression.

## Results

### HilE is necessary for activation of *hilA* expression by acetate

**Fig. 1:**
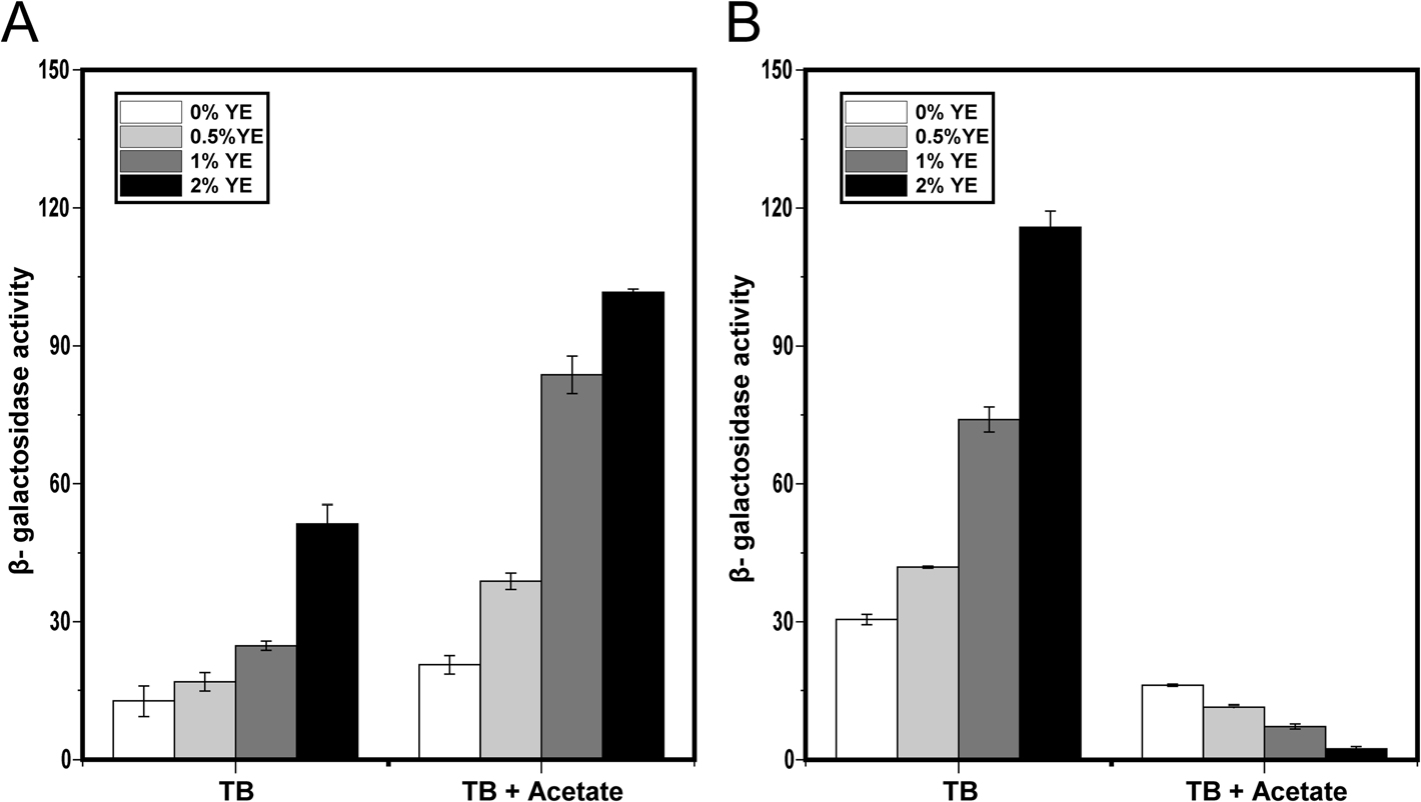
Acetate and yeast extract (YE) synergistically induce *hilA* expression in the wild type (**a**) but not a Δ*hilE* mutant (**b**). Expression was determined by measuring β-galactosidase activity using transcriptional fusions of the *hilA* promoter to LacZ during growth in TB medium with the specified amount of yeast extact added. Sodium acetate was added at a concentration of 10 mM. Results show the mean and standard deviation from three biological replicates

We previously found that during growth on TB medium, the addition of yeast extract and acetate synergistically induce *hilA* expression [13]. HilE negatively regulates HilA expression by binding to HilD and preventing it from activating *hilA* expression [10–12]. Other studies have shown that deleting *hilE* increases *hilA* expression [9]. Therefore, we tested whether yeast extract and acetate would also induce *hilA* expression in a Δ*hilE* mutant. In these experiments, we measured SPI-1 gene expression using transcriptional fusion to β-galactosidase (LacZ) after 12 h of growth, which was chosen for consistency with our previous study and that it yielded strong SPI-1 gene expression [13]. Consistent with these previous studies, we found that *hilA* expression was greater in a Δ*hilE* mutant than the wild type during growth on TB medium (Fig. 1). When yeast extract was added to the growth medium, *hilA* expression increased in both the wild type and Δ*hilE* mutant with expression higher in the Δ*hilE* mutant as expected. However, when acetate was added to the growth medium, *hilA* expression was reduced in the Δ*hilE* mutant even though it increased it in the wild type. The further addition of yeast extract further decreased *hilA* expression (> 2-fold) in the Δ*hilE* mutant.

**Fig. 2:**
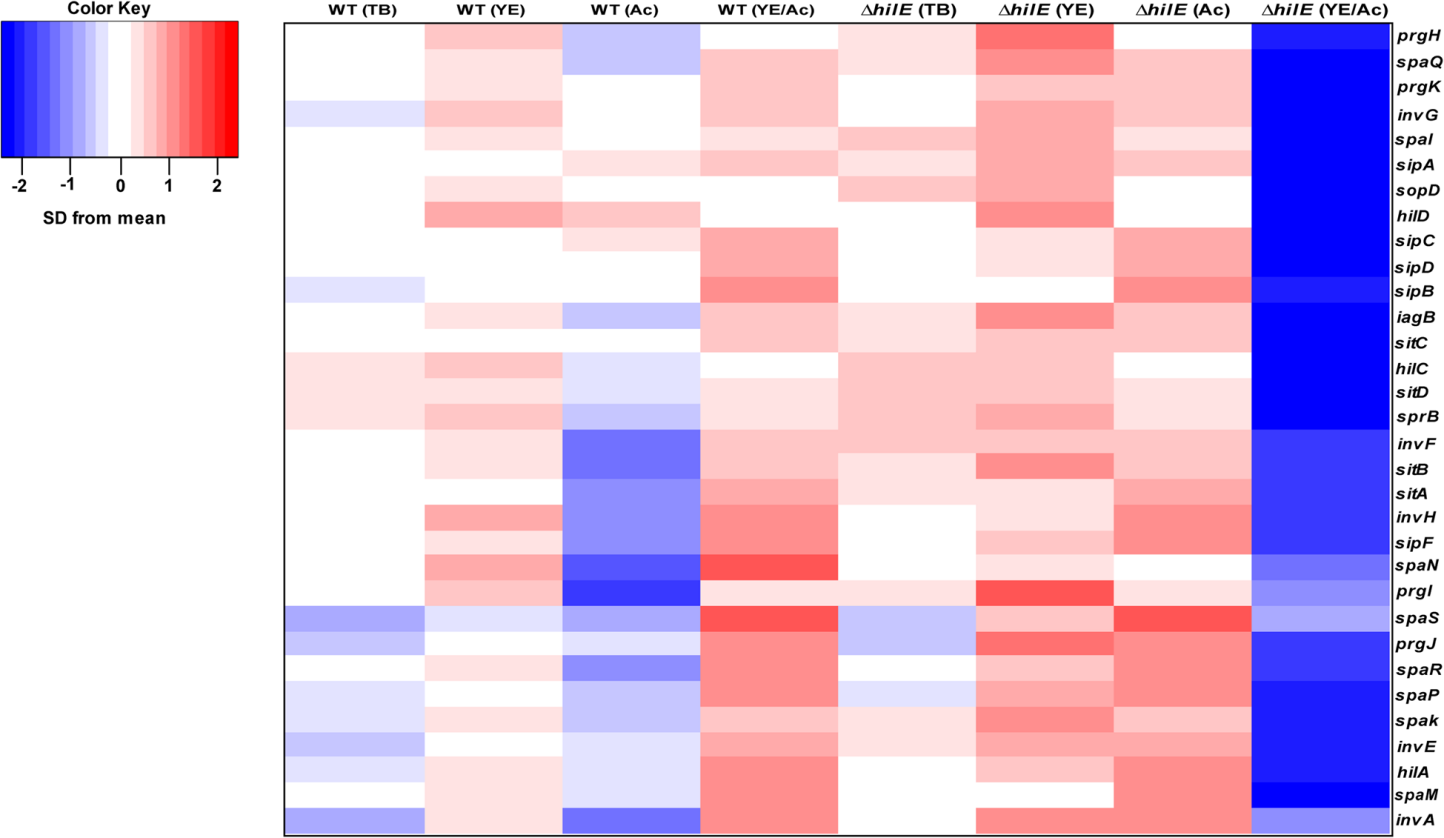
Transcriptional changes associated with the expression of the SPI-1 gene in the wild type (WT) and Δ*hilE* mutant during growth in TB medium, TB medium supplemented with 1% yeast extract (YE), TB medium supplement with 10 mM sodium acetate (Ac), and TB medium supplemented with 1% yeast extract and 10 mM sodium acetate. RNAseq data was collected in triplicate for each condition

In these experiments, we used transcriptional fusions to LacZ. One possibility is that these results are an artifact of using LacZ transcriptional fusions. Therefore, we repeated the same experiments using transcriptional fusions to the green fluorescent protein (GFP), the same reporter used in our previous study [13]. Similar results were observed (Fig. S1). The one exception was that yeast extract weakly increased *hilA* expression (< 2-fold) in the presence of acetate in the Δ*hilE* mutant. As discussed below, we also observed a similar response when profiling gene expression using RNA-Seq (Fig. 2). Collectively, these results suggest that HilE is necessary for activation of *hilA* expression by acetate and that results are not an artifact of the method used to measure gene expression.

### HilE is not necessary for activation of *hilA* expression by other short-chain fatty acids

**Fig. 3:**
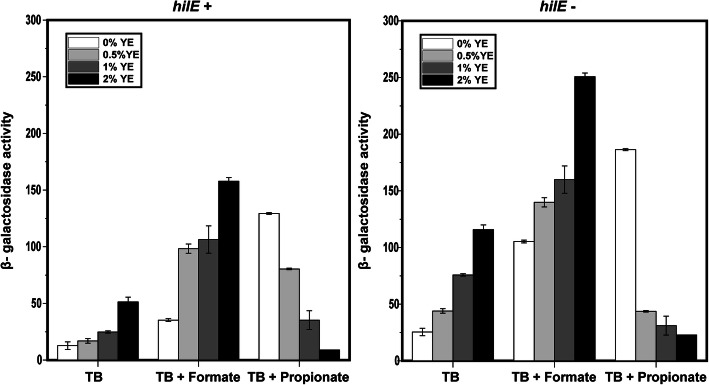
Formate and yeast extract increase *hilA* expression in both the wild type (**a**) and Δ*hilE* mutant (**b**); propionate increases *hilA* expression in both the wild type (**a**) and Δ*hilE* mutant but the further addition of yeast extract (YE) reduces it. Expression was determined by measuring β-galactosidase activity using transcriptional fusions of the *hilA* promoter to LacZ during growth in TB medium with the specified amount of yeast extact added. Sodium formate and sodium propionate were added at concentrations of 10 mM. Results show the mean and standard deviation from three biological replicates

Short-chain fatty acids are known to induce *hilA* expression [14]. Therefore, we tested whether the same response occurred when acetate was replaced with formate or propionate using LacZ transcriptional fusions. Consistent with previous results, we found that formate and propionate induce *hilA* expression (Fig. 3a). In the case of formate, the addition of yeast extract further increased *hilA* expression whereas, in the case of propionate, the addition of yeast extract decreased *hilA* expression. The mechanism governing repression by propionate and yeast extract is not known though the same behavior was observed in the Δ*hilE* mutant as discussed below.

When the same experiments were performed with the Δ*hilE* mutant (Fig. 3b), the same general responses were observed. In the case of formate, expression was higher in the Δ*hilE* mutant than in the wild type, which is consistent with HilE being a negative regulator of *hilA* expression. The addition of yeast extract further increased *hilA* expression. In the case of propionate, the results were mixed. In the absence of yeast extract, propionate increased *hilA* expression in both the wild type and Δ*hilE* mutant. However, in the presence of yeast extract, *hilA* expression was higher in the wild type at some yeast extract concentration and lower at others. These results suggest that loss of HilE has only a minor effect on the response to propionate and yeast extract. Taken together, we conclude that the requirement of HilE for activation of *hilA* expression is specific for acetate though we cannot discount that there is also minor contribution in the case of propionate.

### HilC and RtsA are not involved in repressing *hilA* expression by acetate and yeast extract in a Δ*hilE* mutant

HilD is the master regulator of *hilA* expression. In a Δ*hilD* mutant, *hilA* expression is weak. HilD also activates two additional transcription factors, HilC and RtsA, that can independently activate *hilA* expression. HilC and RtsA appear to amplify *hilA* expression as opposed to HilD, which is necessary for turn *hilA* expression on [8, 22]. There is no evidence to suggest that HilE binds either HilC or RtsA [11]. Nonetheless, we tested whether HilC or RtsA are necessary for repression of *hilA* expression by acetate and yeast extract in a Δ*hilE* mutant. Consistent with what we observed in the wild type, acetate and yeast extract synergistically induced *hilA* expression in Δ*hilC* and Δ*rtsA* mutants (Fig. S2). When *hilE* was also deleted, acetate and yeast extract repressed *hilA* expression in both mutants. These results indicate that neither HilC nor RtsA affect the response to acetate and yeast extract.

### The Bar/SirA two-component system is not involved in repressing *hilA* expression by acetate and yeast extract in a Δ*hilE* mutant

The BarA/SirA two-component system regulates *hilA* expression in response to acetate [14]. It specifically regulates HilD expression through the action of CsrA [15]. Therefore, we tested whether the BarA/SirA two-component system is necessary for repressing *hilA* expression by acetate and yeast extract in a Δ*hilE* mutant. We first compared *hilA* expression in response to acetate and yeast extract in the wild type and a Δ*sirA* mutant (Figure S3). Overall, *hilA* expression was lower in the Δ*sirA* mutant than the wild type, consistent with previous observations. We next compared *hilA* expression in Δ*hilE* and Δ*hilE* Δ*sirA* mutants. Acetate and yeast extract reduced *hilA* expression in both mutants. These results suggest that repression of *hilA* expression by acetate and yeast extract in a Δ*hilE* mutant does not involve the BarA/SirA two-component system.

### Expression profiling suggest that the Rcs is involved in inducing *hilA* expression by acetate and yeast extract

Our results suggest that HilE is necessary for activation of *hilA* expression by acetate and yeast extract under the growth conditions tested. In cells lacking HilE, acetate and yeast extract repress *hilA* expression. These results would naively suggest that HilE is a positive regulator of *hilA* expression. However, HilE is a known repressor of *hilA* expression, and there is no evidence to suggest that it is capable of activating *hilA* expression based on current models. In particular, HilE binds the upstream regulator HilD and prevents it from activating *hilA* expression [10–12]. Therefore, we hypothesized that loss of HilE is somehow affecting the expression of some other regulator of *hilA* expression. To identify such regulators, we profiled transcription using RNA-Seq in the wild type and Δ*hilE* mutant during growth (12 h time point) in TB medium alone, TB medium containing 1% yeast extract, TB medium containing 10 mM sodium acetate, and TB medium containing 1% yeast extract and 10 mM sodium acetate (Table S1). These concentrations of yeast extract and sodium acetate were previously shown to induce *hilA* expression during growth on TB medium.

A total of 522 million raw reads were obtained from 24 samples (3 biological replications for the two strains grown under the four conditions). Approximately 90% of the reads mapped to a unique location on the *S. enterica* genome. During growth on TB medium, we observed 85 genes with increased expression and 25 genes with reduced expression in the Δ*hilE* mutant as compared to the wild type. During growth on TB medium containing 1% yeast extract, we observed 95 genes with increased expression and 241 with decreased expression. During growth on TB medium containing 10 mM acetate, we observed 382 genes with increased expression and 144 with decreased expression. And, during growth on TB medium containing both 1% yeast extract and 10 mM acetate, we observed 457 genes with increased expression and 770 with decreased expression (Fig. S4).

Consistent with the data involving transcriptional fusions for the green fluorescent protein and LacZ, we found that acetate and yeast extract strongly inhibited the expression of the SPI-1 genes involved in the Δ*hilE* mutant relative to the wild type (Fig. 2). Otherwise, the expression of these genes was increased in the Δ*hilE* mutant relative to the wild type as expected. Similar results were also observed with regards to the expression of the flagellar genes, whose expression is coupled with the SPI-1 genes (Fig. S5).

**Fig. 4:**
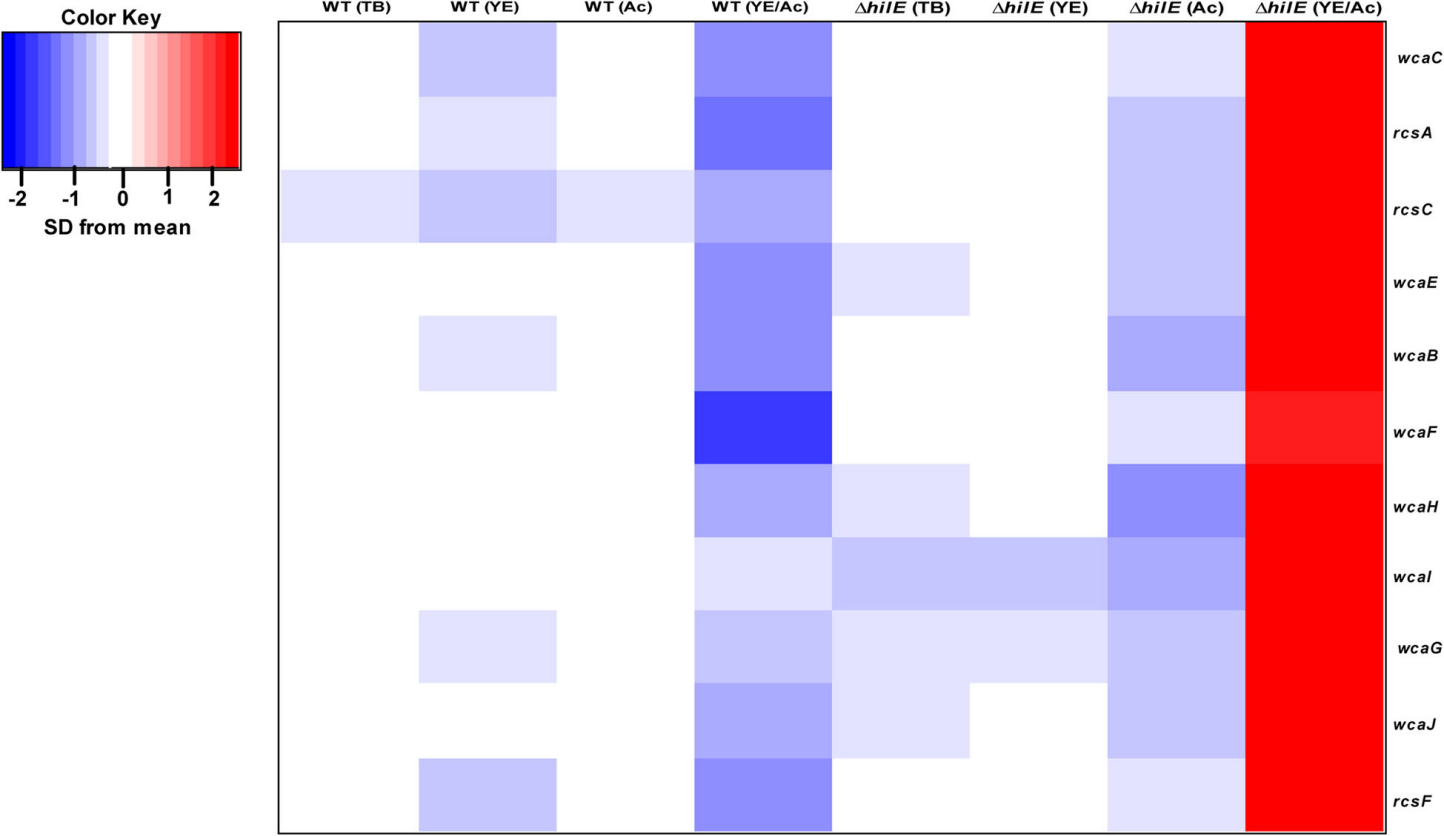
Transcriptional changes associated with the expression of the *rcs* and *wca* gene in the wild type (WT) and Δ*hilE* mutant during growth in TB medium, TB medium supplemented with 1% yeast extract (YE), TB medium supplement with 10 mM sodium acetate (Ac), and TB medium supplemented with 1% yeast extract and 10 mM sodium acetate (YE/Ac). RNAseq data was collected in triplicate for each condition

Among the other differentially expressed genes, we found that acetate and yeast extract activate the expression of genes involved in the Rcs system in a Δ*hilE* mutant (Fig. 4). Consistent with increased expression of the Rcs system, we also observed increased expression of *wca* genes involved in the synthesis of extracellular polysaccharides, which are known to be induced by the Rcs system [23, 24]. Interestingly, the Rcs system is known to repress expression of the SPI-1 genes [25, 26].

### The Rcs system is necessary for activation of *hilA* expression by acetate and yeast extract

**Fig. 5:**
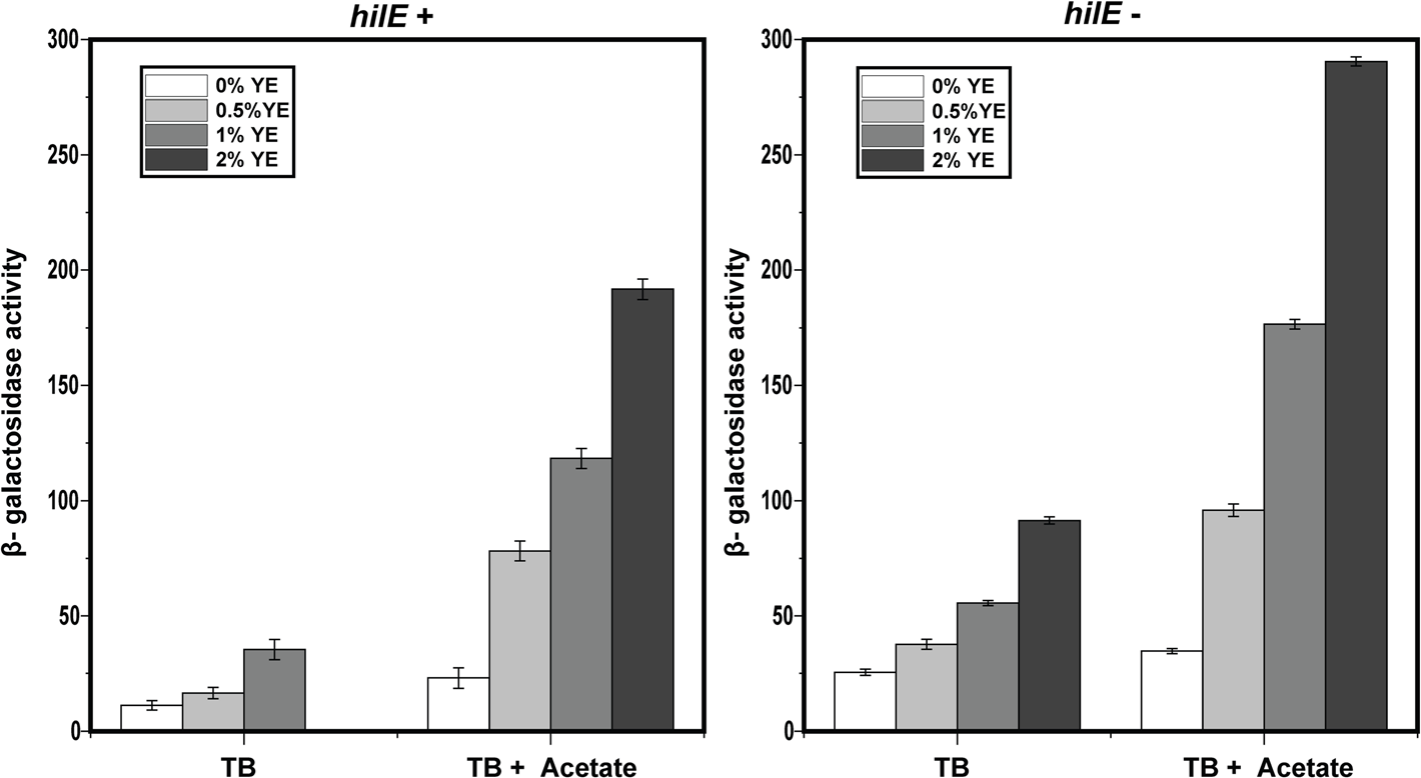
The Rcs system is necessary for repression of *hilA* expression by acetate and yeast extract in a Δ*hilE* mutant. The figures show *hilA* expression in Δ*rcsBC* mutant (A) and Δ*rcsBC* Δ*hilE* mutant. Expression was determined by measuring β-galactosidase activity using transcriptional fusions of the *hilA* promoter to LacZ during growth in TB medium with the specified amount of yeast extact added. Sodium acetate was added at a concentration of 10 mM. Results show the mean and standard deviation from three biological replicates

Analysis of the RNA-Seq data suggest that the Rcs system may govern the response to acetate and yeast extract observed in the Δ*hilE* mutant (Fig. 4). To test this hypothesis, we measured *hilA* expression in Δ*rcsBC* and Δ*rcsBC* Δ*hilE* mutants*,* which lacks the RcsC histidine kinase necessary for signaling (Fig. 5). The response of *hilA* expression to acetate and yeast extract in the Δ*rcsBC* mutant was similar to the wild type, with acetate and yeast extract synergistically inducing expression. Consistent with a model where the Rcs system is necessary for repression of *hilA* expression by acetate and yeast extract in a Δ*hilE* mutant, we again observed that acetate and yeast extract synergistically induce *hilA* expression in a Δ*rcsBC* Δ*hilE* double mutant. These results demonstrate that the Rcs system is necessary for repression of *hilA* expression in the absence of HilE.

## Discussion

HilE is a repressor of SPI-1 gene expression [9, 10]. It is known to bind HilD and prevent it from activating *hilA* expression [11, 12] . HilA, in turn, activates expression of the genes encoding the SPI-1 type III secretion system and associated chaperone and effector proteins. A number of regulators tune SPI-1 gene expression by altering the expression of HilE. For example, LeuO and FimZ repress SPI-1 gene expression by increasing the expression of HilE [27–29] whereas Mlc and the small RNA isrM increase SPI-1 gene expression by decreasing the expression of HilE [30, 31]. All of these mechanisms are consistent with HilE being a repressor of SPI-1 gene expression.

In our work, we found that deleting *hilE* decreases SPI-1 gene expression during growth in TB medium containing acetate and yeast extract. These results suggest that HilE can also function as an activator of SPI-1 gene expression under the specific growth conditions tested, most likely through an indirect mechanism. By analyzing differentially expressed genes in the wild type and Δ*hilE* mutant during growth on different medias, we identified the Rcs phosphorelay system as a critical regulator. Expression of *rcsA*, *rcsC*, and *rcsF* was increased in the Δ*hilE* mutant as compared to the wild type during growth on TB medium containing acetate and yeast extract but not during growth of TB medium containing either acetate or yeast extract. The Rcs phosphorelay system normally represses SPI-1 gene expression in response to envelope stress [25]. Indeed, a Δ*rcsBC* mutant exhibited the same response to acetate and yeast extract. Yet, *hilA* expression was not repressed by acetate and yeast extract in a Δr*csBC* Δ*hilE* mutant; rather, the behavior was similar to the wild type.

We also observed strong repression of the flagellar genes by acetate and yeast extract in the Δ*hilE* mutant (Fig. S5). Previous work demonstrated that synergistic activation of both SPI-1 and flagellar gene expression is due to transcriptional crosstalk between these two systems [13]. Acetate activates SPI-1 gene expression via the BarA/SirA two-component system and yeast extract activates flagellar gene expression via RflP, also known as YdiV. Since gene expression is coupled in these two systems, the net effect is synergistic activation. Not surprisingly then, we also observed repression of flagellar gene expression by acetate and yeast extract.

The mechanism of repression of SPI-1 gene expression by the Rcs phosphorelay system is unknown though part is due to repression of the flagellar genes [25]. In addition, it is not clear whether HilE directly interacts with components of the Rcs system or other proteins affecting its activity. Moreover, in the absence of acetate and yeast extract, SPI-1 gene expression is higher in a Δ*hilE* mutant than in the wild type during growth in TB medium, suggesting the observed behavior is specific to acetate and yeast extract. One possibility is that the response is due to crosstalk between the Bar/SirA and Rcs systems [32]. However, the expressions patterns observed in the Δ*hilE* mutant are not due to the BarA/SirA two-component systems, suggesting some other regulatory system is involved.

**Fig. 6:**
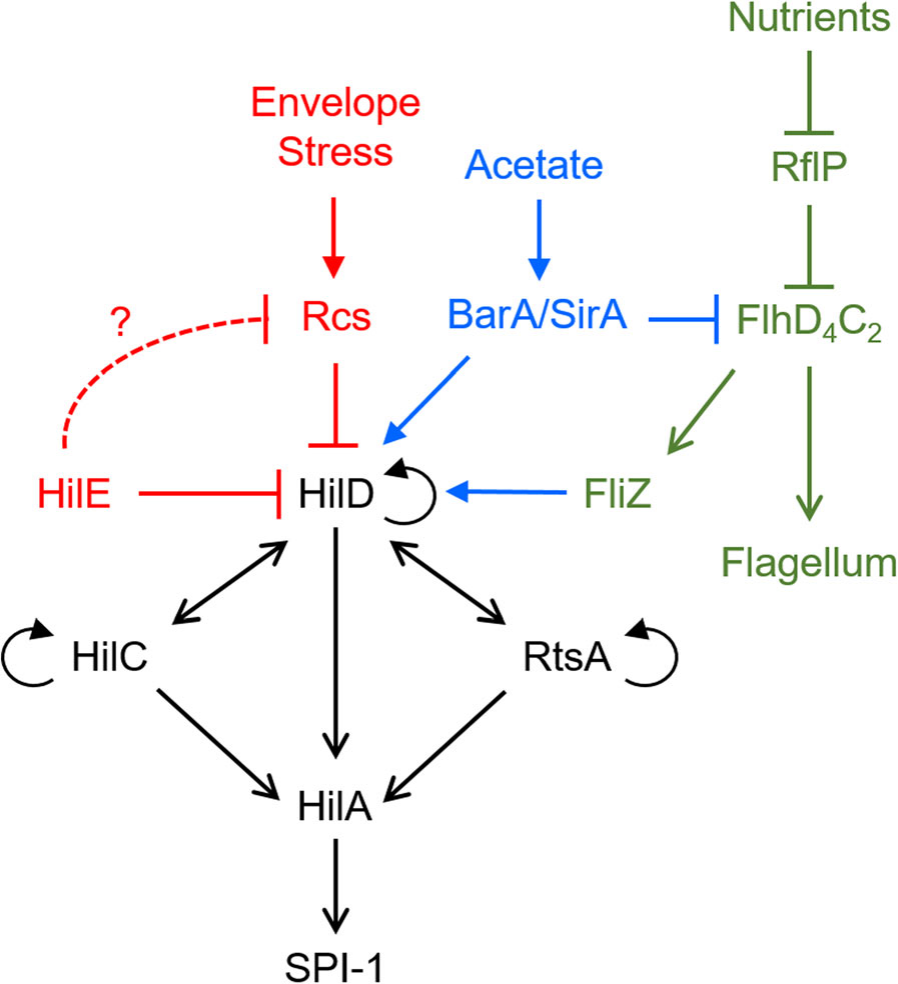
Regulatory network controlling SPI-1 and flagellar gene expression. While the mechanism is unknown, one possibility is that HilE is repressing Rcs mediated repression of SPI-1 and flagellar gene expression. Not shown are repression of flagellar gene expression by the Rcs system and RtsB and activation of flagellar gene expression by HilD

While the mechanism is unknown, our results nonetheless demonstrate that HilE has additional functions to being a repressor of HilD. In particular, HilE is required for activation of SPI-1 gene expression by acetate and yeast extract. At this stage, we have few insights regarding the mechanism beyond the involvement of the Rcs system (Fig. 6). One possibility is that HilE is somehow interacting with specific components of Rcs system, preventing them from repressing SPI-1 gene expression or, alternatively, inducing expression. Indeed, RcsB can associate with other transcriptional regulators [33–36], and perhaps HilE binds these complexes and prevents them from binding their target promoters. In addition, acetate and yeast extract may affect the response of the Rcs system, which nominally responds to envelope stress, via direct phosphorylation or acetylation by acetyl-P [37, 38]. However, the Rcs system has little effect on *hilA* expression in presence of HilE. This would suggest, that HilE perhaps interacts with other proteins that affect the response of the Rcs system. Clearly, more studies are required to elucidate the mechanism.

## Conclusions

We found that HilE is required for activation of SPI-1 gene expression by acetate and yeast extract in *S. enterica*. The mechanism does not involve the BarA/SirA two-component system, which normally induces SPI-1 gene expression in response to acetate. Rather, the Rcs system is involved. Deletion of the Rcs system removes the HilE requirement for SPI-1 activation by acetate and yeast extract. While the exact mechanism is unknown, these results demonstrate the HilE, nominally a repressor of SPI-1 gene expression, can also function as an activator under the growth conditions investigated. Collectively, these results provide new insights regarding SP1 gene regulation and demonstrate that HilE is more complex than initially envisioned.

## Methods

### Bacterial strains and growth conditions

All strains were derived from *Salmonella enterica* serovar Typhimurium ATCC 14028 (American Type Culture Collection) and are listed in Table S2. Mutants were constructed using P22 transduction with previously developed strains as the donor. All growth experiments were perform at 37 °C using tryptone broth (10 g/L tryptone) buffered at pH 7 using 100 mM 3-(*N*-morpholino) propanesulfonic acid (MOPS) as the base medium. Sodium acetate, sodium formate, and sodium propionate were added at concentrations of 10 mM; yeast extract was added at the specified concentrations.

### β-Galactosidase and fluorescence assays

Cells were grown overnight in TB medium and subcultured 1:500 into fresh TB medium containing the specified concentrations of sodium acetate and yeast extract. The cells were then harvested after 12 h growth. The β-galactosidase was performed as previously described using 96-well microplates [39]. Fluorescence was measured using a BD LRS II flow cytometer (BD Biosciences) as previously described [13, 40]. Briefly, we recorded the fluorescence from 50,000–100,000 cells and reported the mean fluorescence. Data extraction and analysis was performed using FCS Express Version 6 (Denovo Software) and to OriginPro 2019. All experiments report the mean and standard deviation from three biological replicates.

### Transcriptomics

RNA samples were isolated from cells, three biological replicates, after 12 h growth. First, the cells were pelleted by centrifugation at 3200×*g* for 10 min and then resuspended in 1 mL of RNA*later* solution (Sigma-Aldrich). The total RNA was then extracted using RiboPure™-Bacteria Kit (ThermoFisher) following the manufacturer’s instruction. The concentration and quality of the extracted RNA were evaluated using an Invitrogen Qubit 4 Fluorometer and a Thermo Scientific NanoDrop One. RNA integrity numbers were determined using an Agilent Bioanalyzer 2100.

RNA samples were sequenced at the Roy J. Carver Biotechnology Center, University of Illinois at Urbana-Champaign. Briefly, ribosomal RNA was removed using the Ribozero Bacteria Kit (Illumina). The RNAseq libraries were prepared with Illumina’s “TruSeq Stranded mRNAseq Sample Prep Kit”. The libraries were quantitated by qPCR and sequenced on one lane for 101 cycles from each end of the fragments on a NovaSeq 6000 using a NovaSeq SP reagent kit. FASTQ files were generated and demultiplexed with the bcl2fastq v2.20 Conversion Software (Illumina).

Adaptors were trimmed from the 3′-end of the reads using Trimmomatic [41] and then analyzed by FastQC [41]. Genome alignments were performed using STAR (STAR/2.7.3a-IGB-gcc-8.2.0) [42] and RNA reads were counted using the featureCounts-subread package. *Salmonella enterica subsp. enterica* serovar Typhimurium (ASM386401v1) was used as the reference genome. FASTA files are available at NCBI (NCBI Accession: PRJNA663059 ID: 663059 SAMN16116301 to SAMN16116324).

Statistical analysis was performed using R (Version 3.6). The edgR function “DGEList” was used to count data obtained from featureCount and the “objectreadDGE” function to calculate the sum counts for each sample, normalized to the library size with low-information genes filtered out. The edgeR-quasi pipeline needs to create a design matrix with one coefficient (column) for each of the eight groups, and growth in TB medium was used as the baseline for comparison and specified the contrast of interest using limma vignette section 9.6. Differential expression was performed using edgeR as follows: the estimateDisp function was used to determine the variance among replicates and perform empirical Bayesian “shrinkage” of variances. Test statistics were calculated using the glmLRT function. Finally, decideTestsDGE function was used to identify differentially expressed genes by setting the *p*-value threshold to be 0.1 and log fold-change to be at least two.

## Supplementary Information

**Additional file 1.**

**Additional file 2.**

## Data Availability

The datasets used and/or analysed during the current study are available from the corresponding author on reasonable request. The RNA-Seq dataset are available at NCBI: https://www.ncbi.nlm.nih.gov/Traces/study/?acc=PRJNA663059.
