# Supplementary material for: HilE is required for synergistic activation of SPI-1 gene expression in Salmonella enterica serovar Typhimurium
Source: BMC Microbiol. 2021 Feb 16;21:49. doi: 10.1186/s12866-021-02110-8 (PMC7887791; doi:10.1186/s12866-021-02110-8)
Supplement: Supplementary file 1 — Additional file 1. [file 12866_2021_2110_MOESM1_ESM.docx]

**SUPPLEMENTARY MATERIAL**

**HilE is required for synergistic activation of SPI-1 gene expression in *Salmonella enteric*a serovar Typhimurium**

Selwan Hamed^1,2^*, Riham M. Shawky^2^, Mohamed Emara^2^, James M. Slauch^3^, and Christopher V. Rao^1^*

^1^Department of Chemical and Biomolecular Engineering, University of Illinois at Urbana-Champaign, Urbana, IL 61801, USA

^2^Department of Microbiology and Immunology, Faculty of Pharmacy, Helwan University − Ain Helwan, Helwan, Egypt 11795

^3^ Department of Microbiology, University of Illinois at Urbana-Champaign, Urbana, IL 61801.

*Corresponding author. Mailing address: Department of Chemical and Biomolecular Engineering, University of Illinois at Urbana-Champaign, 600 S. Mathews Ave., Urbana, IL 61801, USA. Phone: (217) 244-2247. Fax: (217) 333-5052. Email: [cvrao@illinois.edu](mailto:cvrao@illinois.edu)


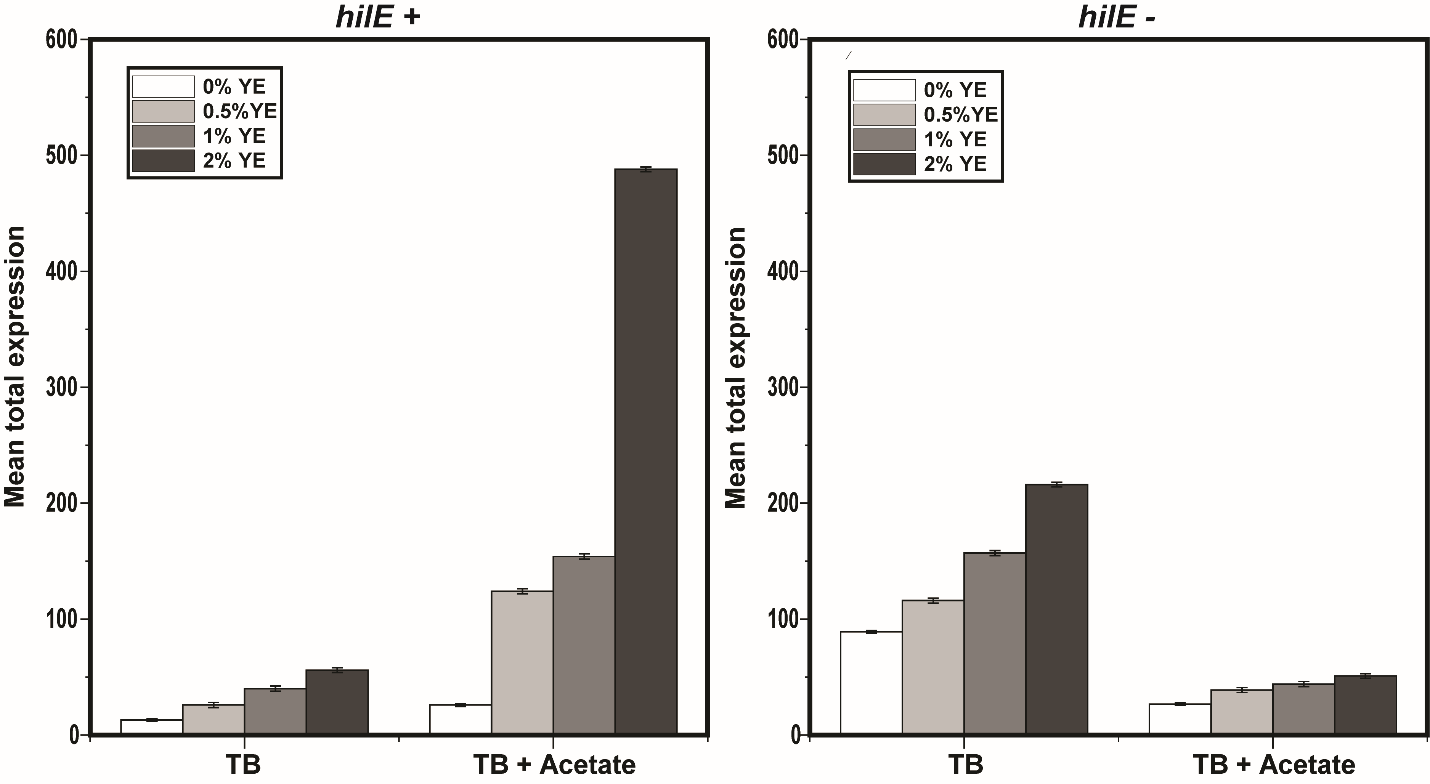


**Figure S1:** Acetate and yeast extract (YE) synergistically induce *hilA* expression in the wild type (A) but not a Δ*hilE* mutant (B). Expression was determined by measuring fluorescence via flow cytometry using transcriptional fusions of the *hilA* promoter to the green fluorescent protein during growth in TB medium with the specified amount of yeast extact added. Sodium acetate was added at a concentration of 10 mM. Results show the mean and standard deviation from three biological replicates.





**Figure S2**. HilC and RtsA do not govern the synergistic response to acetate and yeast extract mediated by HilE. Figure show *hilA* expression in Δ*hilC* (A), Δ*hilC* Δ*hilE* (B), Δ*rtsA* (C), and Δ*rtsA* Δ*hilE* (D) mutants. Expression was determined by measuring β-galactosidase activity using transcriptional fusions of the *hilA* promoter to LacZ during growth in TB medium. Results show the mean and standard deviation from three biological replicates.





**Figure S3.** The Bar/SirA two-component system is not involved in repressing *hilA* expression by acetate and yeast extract in a Δ*hilE* mutant. Expression was determined by measuring β-galactosidase activity using transcriptional fusions of the *hilA* promoter to LacZ during growth in TB medium with the specified amount of yeast extact added. Sodium acetate was added at a concentration of 10 mM. Results show the mean and standard deviation from three biological replicates.


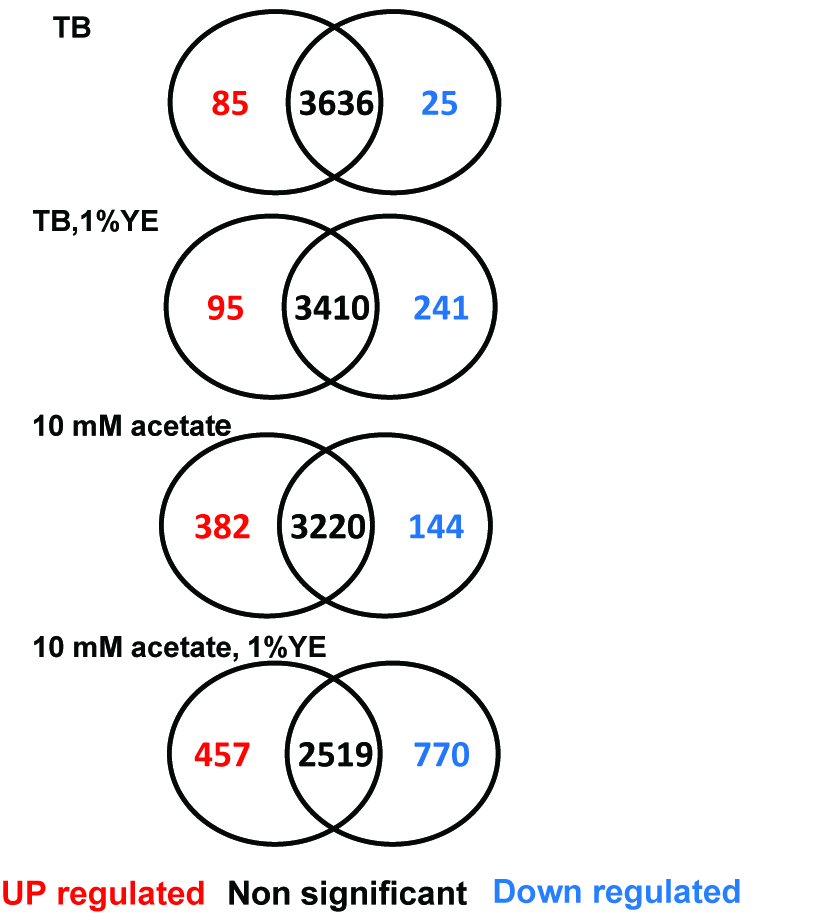


**Figure S4**. Venn diagram shown up and down regulated genes in the Δ*hilE* mutant versus the wild type during growth in TB medium, TB medium supplemented with 1% yeast extract (YE), TB medium supplement with 10 mM sodium acetate, and TB medium supplemented with 1% yeast extract and 10 mM sodium acetate. RNAseq data was collected in triplicate for each condition.


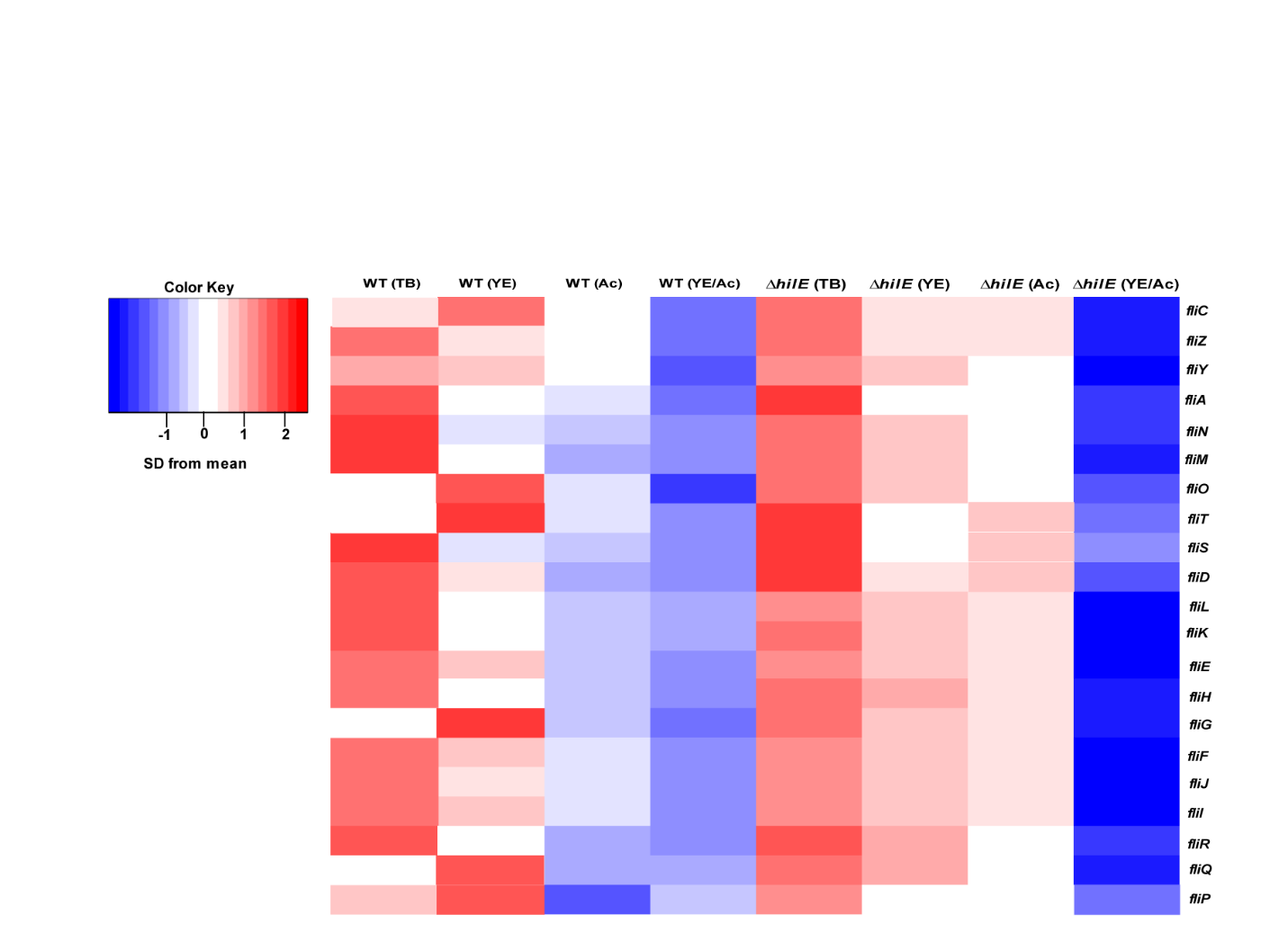


**Figure S5.** Transcriptional changes associated with the expression of the flagellar gene in the wild type and Δ*hilE* mutant during growth in TB medium (TB), TB medium supplemented with 1% yeast extract (YE), TB medium supplement with 10 mM sodium acetate (Ac), and TB medium supplemented with 1% yeast extract and 10 mM sodium acetate (YE/Ac). RNAseq data was collected in triplicate for each condition.

**Table S2: Bacterial strains**

| **Strain** | **Genotype** | **Source** |
| --- | --- | --- |
| 14028 | Wild type | ATCC |
| SH52 | *attλ::P_hilA_-gfp* | [1] |
| SH23 | *ΔhilE::cam attλ::P_hilA_-gfp* | [1] |
| JS749 | *att::pDX1::hilA-lacZ* | [2] |
| JS633 | *ΔhilE::aadR att::pDX1::hilA-lac* | [3] |
| SH67 | *ΔhilE::cat att::pDX1::hilA-lac* |  |
| SH80 | *ΔsirA::cat att::pDX1::hilA-lacZ* |  |
| SH83 | *ΔhilC::frt att::pDX1::hilA-lacZ* |  |
| SH84 | *ΔhilC::frt ΔhilE ::CM att::pDX1::hilA-lacZ* |  |
| SH85 | *ΔrtsA::cat att::pDX1::hilA-lacZ* |  |
| SH86 | *ΔrtsA::cat ΔhilE ::cat att::pDX1::hilA-lacZ* |  |
| SH87 | *ΔhilC::frt ΔrtsA::cat ΔhilE ::cat att::pDX1::hilA-lacZ* |  |
| SH117 | *ΔhilE::cat attλ::PfliC-Venus* |  |
| SH122 | *∆rcsBC::frt λatt::pDx1 hilA'-lacZ* | [4] |
| SH131 | *∆rcsBC::frt ∆hilE::aadR λatt::pDx1 hilA'-lacZ* |  |

All strains are isogenic derivatives of ATCC 14028.

ATCC: American Type Culture Collection

The source was this study, unless otherwise noted.

1. Hamed S, Wang X, Shawky RM, Emara M, Aldridge PD, Rao CV: **Synergistic action of SPI-1 gene expression in Salmonella enterica serovar typhimurium through transcriptional crosstalk with the flagellar system**. *BMC Microbiol* 2019, **19**(1):211.

2. Lin D, Rao CV, Slauch JM: **The Salmonella SPI1 type three secretion system responds to periplasmic disulfide bond status via the flagellar apparatus and the RcsCDB system**. *J Bacteriol* 2008, **190**(1):87-97.

3. Ellermeier JR, Slauch JM: **Fur regulates expression of the Salmonella pathogenicity island 1 type III secretion system through HilD**. *J Bacteriol* 2008, **190**(2):476-486.

4. Palmer AD, Slauch JM: **Envelope Stress and Regulation of the Salmonella Pathogenicity Island 1 Type III Secretion System**. *J Bacteriol* 2020, **202**(17).
